# The prediction of market-level food choices by the neural valuation signal

**DOI:** 10.1371/journal.pone.0286648

**Published:** 2023-06-02

**Authors:** Andrew Kislov, Anna Shestakova, Vadim Ushakov, Mario Martinez-Saito, Valeria Beliaeva, Olga Savelo, Aleksey Vasilchuk, Vasily Klucharev

**Affiliations:** 1 International Laboratory of Social Neurobiology, Institute for Cognitive Neuroscience, HSE University, Moscow, Russia; 2 Decision Neuroscience Lab, Department of Health Sciences and Technology, ETH Zurich, Zürich, Switzerland; 3 Institute for Advanced Brain Studies, Lomonosov Moscow State University, Moscow, Russia; 4 Restart Vasilchuk Brothers, Moscow, Russia; Temple University, UNITED STATES

## Abstract

Neuroimaging studies have demonstrated the ability to use the brain activity of a group of individuals to forecast the behavior of an independent group. In the current study, we attempted to forecast aggregate choices in a popular restaurant chain. During our functional magnetic resonance imaging (fMRI) study, 22 participants were exposed to 78 photos of dishes from a new menu of a popular restaurant chain. In addition to self-reported preferences, fMRI data was extracted from an *a priori* domain-general and task-specific region of interest—the ventral striatum. We investigated the relationship between the neural activity and real one-year sales provided by the restaurant chain. Activity in the ventral striatum, which was defined using the task-specific region of interest, significantly correlated (r = 0.28, p = 0.01) with one-year sales. A regression analysis, which included ventral striatum activity together with the objective characteristics of the products (price and weight), behavioral, and survey data, showed R^2^ values of 0.33. Overall, our results confirm prior studies, which have suggested, that brain activity in the reward system of a relatively small number of individuals can forecast the aggregate choice of a larger independent group of people.

## Introduction

Recent neuroimaging studies have suggested that the brain activity of a group of individuals can forecast the behavior of a separate and independent group of individuals [1–10]. In the current study, we further explore the neuroforecasting approach and test whether the brain activity of a small group of participants can predict the aggregated behavior of costumers in a restaurant chain.

In previous work in this field, a pioneering functional magnetic resonance imaging (fMRI) study revealed a significant correlation between brain activity in the ventral striatum (VS) and the ventral medial prefrontal cortex (vmPFC) with market-level song popularity during the three years following brain scanning [9]. A few neuroforecasting studies used electroencephalography to predict preferences of TV content [8], the popularity of YouTube videos [10], the subsequent choices of consumer products from the online website [11], and the commercial success of movie trailers [7]. However, the most popular neuroforecasting method at present is fMRI due to its sensitivity to subcortical activity [12]. Previous fMRI neuroforecasting studies successfully used VS activity to forecast the popularity of songs [9], microlending success [3], effectiveness of advertisements [1, 2], internet video popularity [5], point-of-sales materials efficiency [6], viral marketing success [13], article forwards [14], and funding success [4].

According to a “partial-scaling” account, only a certain fraction of neurocognitive processes underlying individual choices can effectively forecast aggregate choice [12]. The affect-integration-motivation framework has proposed a gradient of choice generalization: higher order integrative circuits promote choice consistency within individuals, while lower order affective circuits might more broadly generalize to forecast aggregate choices [15]. Hence, recent neuroforecasting studies have particularly focused on the key region of affective processing—the VS in forecasts of real aggregate choice data [2–4, 9]. A few previous studies have suggested that the activity of the VS can be best generalized to forecast aggregate choice of goods (e.g., [3, 9]). For example, an fMRI study found that although both VS and vmPFC activity predicted individual choices, only VS activity successfully predicted aggregate choices [4]. Importantly, the VS activity often shows the highest valuation-related meta-analytic statistics (e.g., [16]), valuation encoding of the chosen option is often stronger and starts earlier in the VS than in vmPFC (e.g., [17]). Therefore, in the current study, we have focused particularly on the neuroforecasting ability of VS activity.

Interestingly, certain neuroforecasting studies have examined the activity in the same predefined region of interest (ROI) at the VS to forecast a wide variety of aggregate choices, from video engagement in an internet attention market [5] to microlending decisions [3] and stock market price dynamics [18]. Wide popularity of the predefined ROIs implies, in some way, a domain-general role of the VS in reward processing. Nevertheless, here, we raise the question regarding the domain specificity of VS activity forecasting actual population-level choices.

To select among numerous kinds of rewards, organisms require a single common currency of valuation in order to compare options. According to dominant decision theories, organisms use a single common currency to assign values to each alternative, and they subsequently select the alternative with the highest value [19]. Various neuroimaging studies have suggested that the VS encodes subjective values in a single common currency for various types of rewards (for a review, see [16, 20]). Notably, the VS encodes both anticipatory and actual rewards in multiple domains (including primary and secondary rewards), which is similar to a domain-general common currency signal. The idea of a domain-general common (neural) currency implies that both primary rewards (milk, sugar, etc.) and secondary rewards (money, number of likes on social media, etc.) should similarly activate the VS and vmPFC [16, 20–24]. Studies on human subjects have predominantly used *secondary rewards such as money*. Nevertheless, a meta-analysis of 87 fMRI studies compared activations to monetary, erotic, and food reward outcomes [25]. All reward types of the rewards activated the vmPFC, ventral striatum, amygdala, anterior insula, and mediodorsal thalamus but with some differences in the location of peak activity. Another meta-analysis of 206 fMRI studies on rewards also reported overlapping activations for primary and monetary rewards [16]. Recently, a novel well-controlled paradigm demonstrated that increasing amounts of a primary reward and secondary reward can activate the VS and the vmPFC, but a primary reward more strongly activates the right VS compared with secondary rewards [26]. Another seminal study showed partially distinct activations representing the subjective values of monetary and food items: the vmPFC and VS represented the subjective values of both reward types, but only the vmPFC significantly represented the subjective value of money and food on a common scale [23]. Furthermore, an animal study showed that the inactivation of the VS using tetrodotoxin blocks the effect of primary reward but not of the secondary rewards effect [27].

Importantly, an activation likelihood estimation meta-analysis of fMRI studies of the Monetary Incentive Delay Task identified consistent reward anticipation activity particularly in the right VS [28]. Furthermore, in a seminal study, Levy and Glimcher [23] demonstrated the peak activity for the expected subjective value of food in the right VS, while activity for the expected subjective value of money peaked in the left VS. Importantly, neuroimaging studies have implicated the right VS in eating disorders [29] or have shown a stronger right VS activity related to behavioral preferences for food flavors [30]. Since different reward types are processed by partially distinct valuation networks, in the current study, in addition to the whole brain analysis, we particularly focused on the ability to neuroforecast aggregate food choices based on the activity of the right VS.

Here, we further explore whether domain-general ROIs can be universally employed to forecast the behavior of independent groups and whether domain- or task-specific ROIs are required. To neuroforecast aggregate choice in a popular restaurant chain, we used three different ROIs: (i) the ROI specified using the previous meta-analysis of fMRI studies examining neural correlates of subjective value [16], (ii) the ROI specified using the local maximum from a previous neuroforecasting study of secondary rewards [3], and (iii) the ROI specified using the functional localizer for preference-related activity in the current study. Importantly, a domain-general neuroforecasting account would suggest that VS activity in the ROI specified using meta-analysis or previous neuroforecasting studies should better forecast aggregate choices than VS activity in the ROI specified by the domain-specific functional localizer. The opposite would be true for a domain-specific neuroforecasting account. Therefore, in the current study we compared neuroforecasting power of domain-specific and domain-general ROIs.

Thus far, neuroforecasting studies mainly have focused on secondary rewards. The only neuroforecasting study of a primary reward focused rather on marketing communications (for a single chocolate bar) than on the primary reward per se [6]. To the best of our knowledge, no single study has investigated the possibility of neuroforecasting aggregate choices of a variety of primary rewards, such as food or drinks. Therefore, in the current study, we have attempted to neuroforecast aggregate choices of various dishes in a popular restaurant chain. During the fMRI study, the participants were exposed to photos of dishes from the new menu of a popular restaurant chain. The fMRI data was extracted from the right VS and correlated with follow-up one-year sales provided by the restaurant chain. Overall, our results confirmed that brain activity can forecast behavior of a group of people at the market level.

## Method

### Participants

Potential participants (n = 372) were recruited via social media. After receiving a response from a potential participant, the participant had to meet the following criteria to be selected: no history of psychotropic drugs, no substance abuse in the past month, and no history of neurological disorders. Second, to fit the restaurant chain’s target audience, people with the following additional criteria were selected: age = 20–40 years, ownership of high-grade home appliances, ownership of a car or real estate, an average check of at least 1500 monetary units (⋍ 65 USD, according to the Big Mac Index) at this target restaurant chain, and at least one visit to the restaurant chain within the past three months. Thereafter, 27 right-handed participants were randomly selected from the participant pool and invited to participate in an fMRI session. The invitations were sent via email (with an acceptance rate ⋍ 7.5%). All participants provided written informed consent and were unaware of the main purpose of the study. All research protocols were approved by the local university Committee on Interuniversity Surveys and Ethical Assessment of Empirical Research. All human subjects gave their informed verbal consent prior to their participation, and adequate steps were taken to protect the participants’ confidentiality.

### Power analysis and sample size

Because our study focused on aggregate choices (global sales)—as opposed to the prediction of individual choices (in the scanner)—we averaged behavioral and neural data across participants so that the stimulus (i.e., dish as opposed to participant) served as a fundamental unit of analysis. Thus, the stimulus “sample size” was determined using the same approach as done in similar studies using fMRI data to predict population behavior [3, 9]. Based on the previously established estimation procedures [31], a stimulus effect size of 72 units (dishes in our case) would be required to achieve a power of 0.80 at an alpha level of 0.05. Thus, the stimuli in the form of 78 dishes were included in the “food choice task.”

The “participants” sample size was also estimated via previously established procedures [31]; approximately 21–23 participants would provide a power of 0.80 at a conservative brain-wide alpha threshold of 0.002 with *μ*_D_ = 0.5, *σ*_B_ = 0.5%, and *σ*_W_ = 0.75%; however, such thresholds must be relaxed for detecting activity in regions where an effect is predicted (for the same approach, see [3]). The final data analysis included 22 participants.

### Study design

During the fMRI session, each participant was exposed to colored photos of dishes, the names of the dishes, and the prices of the dishes (food choice task). At the end of each trial, the participant accepted or rejected the dish, knowing that one of their choices would be randomly selected at the end of the session and that the dish would be provided. During the survey session, the participants would then answer questionnaires that further screened their preferences for the dishes.

**Fig 1:**
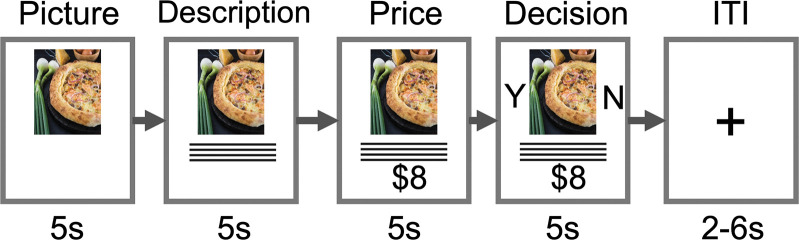
Neuroimaging task: Trial structure. Participants observed an image of a dish (5 s), the name of the dish, and a short description of the dish (5 s). At the end of the trial, participants indicated their choice (5 s), followed by a variable fixation interval (2 s-6 s).

The “food choice task” imitated food selection in a restaurant (Fig 1). Participants were instructed that they would be exposed to the excerpts from the real menu of the well-known popular restaurant. The trial structure was adapted from the study of Genevsky and Knutson (2015). During each trial of the task, participants initially viewed a photograph (for 5 s); the next screen presented the name of the dish and provided details of the contents of the dish (for 5 s). All photos and information were taken from an actual menu of the restaurant, including the prices and weight of each dish (‘objective characteristics’). At the end of each trial, participants indicated whether or not they would eat this dish at the end of the study (5 s). The selected option was highlighted until the end of the trial. The left/right position of the “yes” and “no” prompts was counterbalanced across participants. At the beginning of each trial, the participants fixated on a cross during a variable intertrial interval (2–6 s) A total of 78 trials (78 different dishes from 8 food categories: desserts, main dishes, and appetizers, mean price = 416 monetary units, sd = 139) were randomly presented for approximately 35 minutes. The dishes (Greek salad, beef stroganoff, pizzas, etc.) belonged to European, Middle Eastern, Eastern European, and local cuisine. The complete list of dishes is presented in S13 Table in S1 File.

The participants were instructed to refrain from eating for three hours before the study. At the beginning of the study, the participants were informed that one of their choices (dish) during the “food choice task” would be selected at random and that they would receive a free voucher to consume this dish. Therefore, at the end of the study, the participants received a voucher and consumed the dish of their choice later during a visit to one of the restaurants of the restaurant chain.

Based on the participant’s in-scanner responses, for each dish, we calculated the *in-scanner choices index*, which represented the percentage of participants who indicated their buying intention for the dish during the fMRI session.

After the fMRI session, participants moved to another experimental room, where they rated the same food items and answered the following questions:

1. “Do you like it?” using a five-point Likert scale from 1 (“not at all”) to 5 (“very much”)
2. “Are you satisfied with the price of this dish?” using a five-point Likert scale from 1 (“not at all”) to 5 (“very much”)
3. “Have you ever tested this dish?” (Yes/No)

Based on this data, we calculated several survey indexes for each dish: (1) *likeability–*percentage of people, who selected a rating of more than “3” on the 5-point “Do you like it?” scale; (2) *price perception–*percentage of people who selected a rating of more than “3” on the 5-point “Are you satisfied with the price of this dish?” scale; (3) *familiarity–*percentage of people who answered “Yes” to the question, “Have you ever tested this dish?”.

The descriptive statistics are presented in S2 Table in S1 File. After completing the survey session, a participant received a monetary reward for participation (*currency will be identified if the article is published* or according to Big Mac Index ⋍ 40 USD) and an additional monetary bonus to order (buy) the dish “purchased” during the experiment, depending on the dish price.

### Sales data

**Fig 2:**
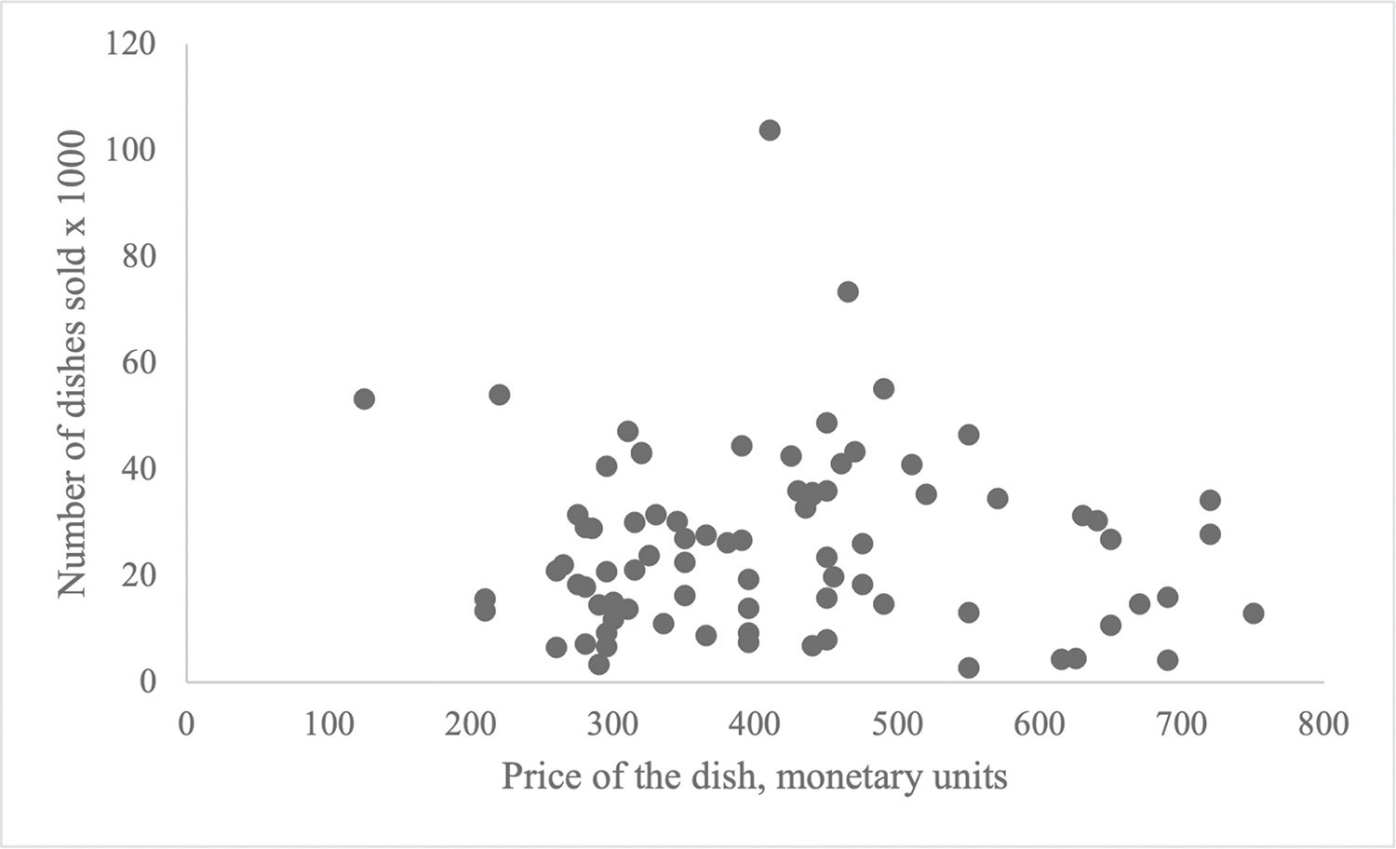
One-year sales (sales index) of 78 dishes within seven price ranges.

The restaurant chain includes 47 restaurants that are part of a large restaurant holding nearly 80 restaurants. These restaurants focus on casual dining ($ $–$ $ $) with an average check of $65–80 according to the Big Mac Index, offering vegetarian-friendly European, Middle Eastern, Eastern European, and local cuisine. Overall, the restaurants offer a menu with nearly 40 appetizers, 90 main dishes, and 10 desserts. After the neuroimaging part of the study was completed, the restaurant chain provided data on the actual number of sales per dish for each day of the 12-month period. Correlational analysis showed that weekly sales for all dishes were highly correlated (r>0.95). Therefore, for each product, we calculated a *sales index* as an aggregate of sales for 12 months (total number of sales). Fig 2 illustrates the distribution of the *sales index*.

### fMRI data acquisition

Functional MRI data was collected using ascending interleaved slice acquisition with gradient echo T2*-weighted echo-planar imaging (EPI) sequence in a 3T Magnetom Verio equipped with a 32-channel head coil (Siemens; Erlangen, Germany). The following values made up the scanning protocol parameters: TE = 30 ms; flip angle = 80°; TR = 2280 ms; slice thickness = 3 mm; no gap; slice matrix = 80 × 80; number of axial slices = 39; FoV = 200 mm; Voxel resolution = 2.5 × 2.5 × 3 mm.

For anatomical localization, we collected high-resolution structural MRI data acquisition using a T1-weighted MP-RAGE sequence. The following parameters were used: TE = 1.76 ms; flip angle = 9°; TR = 1470 ms; slice thickness = 1 mm; slice matrix = 512 × 512 × 176; number of slices = 176; FoV = 320 mm; voxel resolution = 1 × 1 × 1 mm. The scanner performed a corrective routine aimed at counteracting susceptibility angled through the slice plane (z-shimming). The slice angle was tilted at negative 30° with respect to the anterior commissure-posterior commissure axis in the sagittal plane in order to reduce the unaccounted spatial components of the susceptibility gradients [32]. Another reason for this is due to the fact that it allows for better acquisition of the orbitofrontal cortex [33]. The number of volumes acquired was, on average, 830, which corresponded to a duration of approximately 35 minutes. Human subjects were recruited and scanned at a location which will be identified if the article is published.

### Data analysis

#### fMRI preprocessing

We performed preprocessing, first-, and second-level analysis using SPM12 (Wellcome Department of Cognitive Neurology, Institute for Neurology, London, England). The preprocessing included several stages. First, we sinc-interpolated voxel time series to correct for non-simultaneous slice acquisition within each volume; this was corrected for motion, and slightly spatially smoothed to minimize the effects of anatomical variability (4-mm full-width/half-maximum kernel). This time series was then high-pass filtered (admitting frequencies with periods < 90s) and normalized to percentage signal change with respect to each voxel’s average over the entire task. Because of excessive head movements (> 2.0 mm in any dimension from one volume acquisition to the next), we excluded five participants from our sample.

#### Regionally targeted analysis

The preference (localizer) whole-brain analysis included two event-related parametric regressors of interest: (1) the onset of pictures (dishes) that were highly liked (ranked more than three points on the five-point Likert scale), and (2) the onset of pictures (dishes) that were disliked (ranked below three points on the five-point Likert scale). These preference-related data were collected separately from the main study behavior task. The analyses also included eight regressors of no interest. Six of the regressors indexed head motion parameters estimated from the fMRI realignment preprocessing step, and two indexed signals associated with cerebrospinal fluid and white matter intensity [34]. Prior to inclusion in the models, the regressors of interest were convolved with a single gamma-variate function that modeled a canonical hemodynamic response [35].

To localize preference-related neural activity, the following *dish preference* contrast was calculated: activity evoked by the onset of pictures (dishes) that were highly liked *vs*. activity evoked by the onset of pictures (dishes) that were disliked (for a similar approach, see work [36]). The only significant cluster within the striatum (thresholded at p < 0.001) was observed in the right VS (MNI coordinates [18, 8, 2]). For the details on the analysis of choice- and price-related brain activity, see S1 File.

### Targeted neuroforecasting analysis

#### Correlation analysis

Targeted ROI analysis was conducted in a manner similar to previous neuroforecasting papers [3]. First, we selected ROIs based on previous meta-analyses and neuroforecasting studies [3, 16]. All coordinates were converted to an MNI space (for details, see Table 1). The center of the first ROI, hereinafter referred to as *meta-analyses-based ROI* [12, 10, –6], was selected according to the coordinate-based meta-analyses that examined the neural correlates of the valuation system [16]. The center of the second ROI, hereinafter referred to as *neuroforecasting study-based ROI* [11, 15, –6], was specified using the local maximum from a previous neuroforecasting study [3]. The third ROI (hereinafter referred to as the *functional ROI*) was specified using the local maximum of activation in *dish preference* contrast, and spherical volumes of interest (4 mm in radius) were placed in the right VS (MNI coordinates) [18, 8, 2]. Brain activity was averaged over the first two brain volume acquisitions of each trial, i.e., during presentation of the dish photograph, lagged by 4s to account for the hemodynamic delay (for a similar approach, see [3]). For the main analysis, the ROI activity (percentage signal change) was averaged within each ROI and then extracted to derive the time courses of activation. For extraction, we utilized adapted MATLAB scripts from the MarsBaR toolbox (http://marsbar.sourceforge.net/). Importantly, for each ROI brain activity was averaged across participants. Next, we conducted Pearson’s correlation analysis of the relationship between the *sales index* and the right VS activity (percentage signal change) during the initial presentation of dishes (the first time-window of the trial, “Picture,” see Fig 1. For the primary analysis, we used the “Picture” stage because pioneering neuroforecasting studies particularly have focused on the neural response to initial stimuli presentation: on the initial listening period [9] for songs; project description [4]; and photos of consumer products (cars) [37]. Additionally, S8 Table in S1 File shows that the percent signal change values of the fMRI signal during the “Picture” stage was particularly sensitive to the *dish preference* contrast.

**Table 1:** Correlation of the VS activity during the initial presentation of the dishes (“Picture” stage of the trial) with *sales index* (aggregate sales).

	ROI	MNI coordinates	Correlation with *sales index* (r)	p-value
*Functional ROI*	Right VS (based on the localizer)	18 8 2	0.28	0.01
*Meta-analysis-based ROI*	Right VS (based on the meta-analysis [16])	12 10–6	0.20	0.06
*Neuroforcasting study based ROI*	Right VS (based on the previous study [3])	11 15–6	0.15	0.19

#### Linear mixed regression models

Using linear mixed regression models, we examined whether in-scanner behavior (choices), survey data, objective characteristics of dishes, and neural activity collected during the neuroimaging task could determine behavior at the population level, or *sales index*. Each predictor, except objective characteristics, was standardized and normalized within subjects (z-score transform), and then averaged across subjects. In-scanner choices, survey data, and neural activity were averaged across participants and were treated as fixed effects. Dishes were grouped into eight dish categories and these dish categories were treated as random effects. We used the aggregate *sales index* as the dependent variable and up to seven predictors, including dish categories. The following eight models were tested to explore various predictors of the *sales index* (for supplementary regression models with a larger number of predictors, see S1 File):

1. *Model I* (null model) included only objective characteristics of the dishes (price and weight of each dish) as predictors.
2. *Model II* included participants’ choices (whether or not participants chose the dish during the fMRI session) as predictors.
3. *Model III* included survey data (participants’ preferences: such as whether participants liked the dish, were familiar with the dish, and considered the dish’ price high or low) as predictors.
4. *Model IV* included VS activity (at the *functional ROI*) averaged over the first two brain volume acquisitions of each trial (i.e., during presentation of the dish photograph, lagged by 4s to account for the hemodynamic delay) as a predictor.
5. *Model V* included all the above-mentioned variables.
6. *Model VI* included all the above-mentioned variables except VS activity.
7. *Model VII* was similar to *Model VI*, but instead of the *functional ROI*, it included neural activity at the ROIs (VS, anterior insula, amygdala, and orbitofrontal cortex) as defined based on a previous neuroforecasting study [3].
8. *Model VIII* was similar to *Model VI*, but instead of the *functional ROI*, it included neural activity at the ROIs (VS and vmPFC) as defined based on the previous meta-analysis [16].

Additionally, we used the same approach to examine, which variables could determine *in-scanner choices index*:

1. *In-scanner Model I* (null model) included only the objective characteristics of the dishes (price and weight of each dish) as predictors.
2. *In-scanner Model II* included the participants’ choices (whether or not participants chose the dish during the fMRI session) as predictors.
3. *In-scanner Model III* included survey data (participants’ preferences, such as whether the participants liked the dish, were familiar with the dish, and considered the dish’s price high or low) as predictors.
4. *In-scanner Model IV* included VS activity (at the *functional ROI*) averaged over the first two brain volume acquisitions of each trial (i.e., during presentation of the dish photograph, lagged by 4 s to account for the hemodynamic delay) as a predictor.
5. Combined *in-scanner Model V* included all the above-mentioned variables.

Model comparison results using likelihood ratio tests are presented in Table 4. Additional models for the *in-scanner choices index* are presented in S4-S5 Tables in S1 File. Overall, the models included *dish price*, *dish weight*, *choice in scanner*, *likeability*, *familiarity*, *price perception* and *brain activity* as fixed effects and *dish category* as random effect.

## Results

Five participants were excluded from the analysis because of excessive head movements during scans (i.e., > 2 mm from one scan acquisition to the next). Thus, the final data set included s participants (14 females; mean age = 36.2, sd = 6.1). During the decision stage of the task, the participants selected 34.9% of dishes to be able to receive one of them after the study. Overall, the participants liked 79.5% of the dishes (for more details, see S2 Table in S1 File).

### Correlation analysis

**Fig 3:**
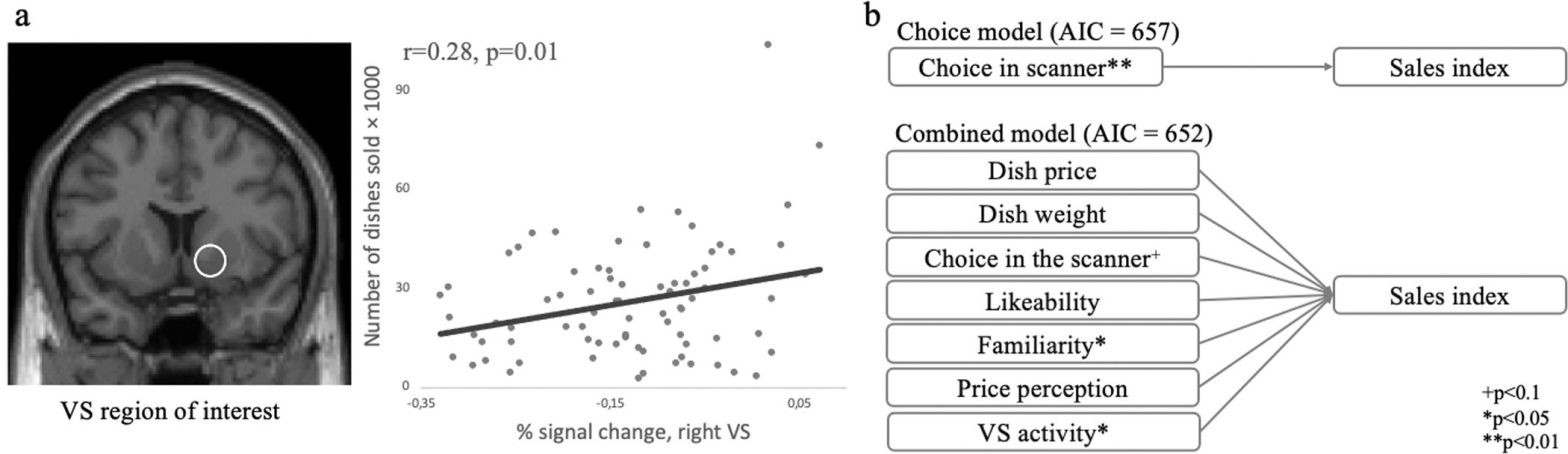
Relationship among VS activity, choices, survey data, dishes’ objective characteristics, and *sales index*, which indicates population behavior. (a) The ROI-1 and the scatterplot indicate *sales index* as a function of averaged VS activity. The line represents a linear trend estimate. (b) The diagrams show key results of linear regression models that estimated whether objective characteristics (dish’s price and weight), choices, survey metrics (likeability, familiarity, price perception), and VS activity were predictive of *sales index*. Akaike information criterion value is given in parentheses; asterisks indicate significant coefficients (*p < .05, **p < .01).

Following previous neuroforecasting studies [3], we calculated the correlations between *sales index* and activity in predefined *ROIs* at the VS during the initial presentation of the dishes (“Picture” stage of the trial). We found no significant correlation between the *sales index* and the grand average VS activity at the *meta-analysis-based ROI* (r = 0.2, p = 0.06) or *neuroforecasting study-based ROI* (r = 0.15, p = 0.19). However, the averaged VS activity at the *functional ROI* significantly correlated with the *sales index* (r = 0.28, p = 0.01, see Fig 3A). The results are summarized in Table 1.

A supplementary analysis of the in-scanner behavior showed a not significant correlation between the *in-scanner choices index* and VS activity at the *functional ROI* (r = 0.18, p = 0.09; S3 Table in S1 File). Similarly, we found no significant correlation between the *in-scanner choices index* and the *meta-analysis-based ROI* or *neuroforecasting study-based ROI*: r = 0.02, p = 0.87 and r = 0.08, p = 0.46, correspondingly (for details, see S3 Table in S1 File).

Hierarchical linear regression modeling for (aggregate) sales index

Separate linear regression models estimated whether (a) objective characteristics (dish price and weight), (b) in-scanner behavior (choices), (c) survey metrics (likeability, familiarity, price perception), and (d) VS activity (*functional ROI*) were predictive of behavior at the population level (see Table 2 and Fig 3B). The Akaike information criterion (AIC) indicated a better fit of combined *Model V*, which combined all terms (AIC = 652) compared with *Model I* (AIC = 663), *Model II* (AIC = 657), Model III (AIC = 654) or *Model IV* (AIC = 658), despite penalties for additional predictors (see Fig 3B). Conditional and marginal adjusted R^2^ are reported in Table 2 and describe the proportion of variance in the *sales index* as explained by the independent variables; it indicates that Model V has the better fit, than other models.

**Table 2:** Results of the linear regression models predicting *sales index* using objective characteristics of dishes (price, weight), in-scanner behavioral, survey, or/and neuroimaging data for the *functional ROI*.

Predictor	Model				
	Objective characteristics (Model I)	In-scanner choices (Model II)	Survey data (Model III)	Brain activity (Model IV)	Combined (Model V)
*Intercept*	25.62(7.10)**	26.90(3.48)**	26.69(2.97)**	27.00(2.92)**	19.61(10.85)+
*Dish price*	-0.02(0.01)				-0.01(0.02)
*Dish weight*	0.03(0.02)				0.03(0.02)
*Choice in scanner*		15.82(5.86)**			15.21(9.23)^+^
*Likeability*			13.77(5.68)*		4.97(7.99)
*Familiarity*			-17.67(8.20)*		-15.55(7.79)*
*Price perception*			-0.57(2.97)		0.85(5.80)
functional ROI:					
*Right VS* ^ⅰ^				18.94(7.82)*	19.42(7.06)**
R^2^ marginal	-0.02	0.04	0.12	0.08	0.17
R^2^ сonditional	0.15	0.21	0.24	0.17	0.33
AIC	663	657	654	658	652

^ⅰ^ functional ROI

The table presents standardized coefficients with standard error in brackets. +indicates p-value<0.1; *indicates p-value<0.05

**indicates p-value<0.01

We also tested combined regression models that included different ROIs. *Models V*, *VII*, and *VIII* explained the *sales index* more than *Models VI*, which omitted fMRI data, both in terms of the AIC, conditional and marginal adjusted R^2^. Results of this analysis are presented in Table 4.

A combination of VS activity (*functional ROI*), in-scanner choices, participants’ preferences, price perception, and objective product characteristics explained 33% of the variance in aggregate population behavior indicated by the *sales index*. VS activity alone explained 17% of the variance in the *sales index*.

### Linear regression modeling for in-scanner choices

**Fig 4:**
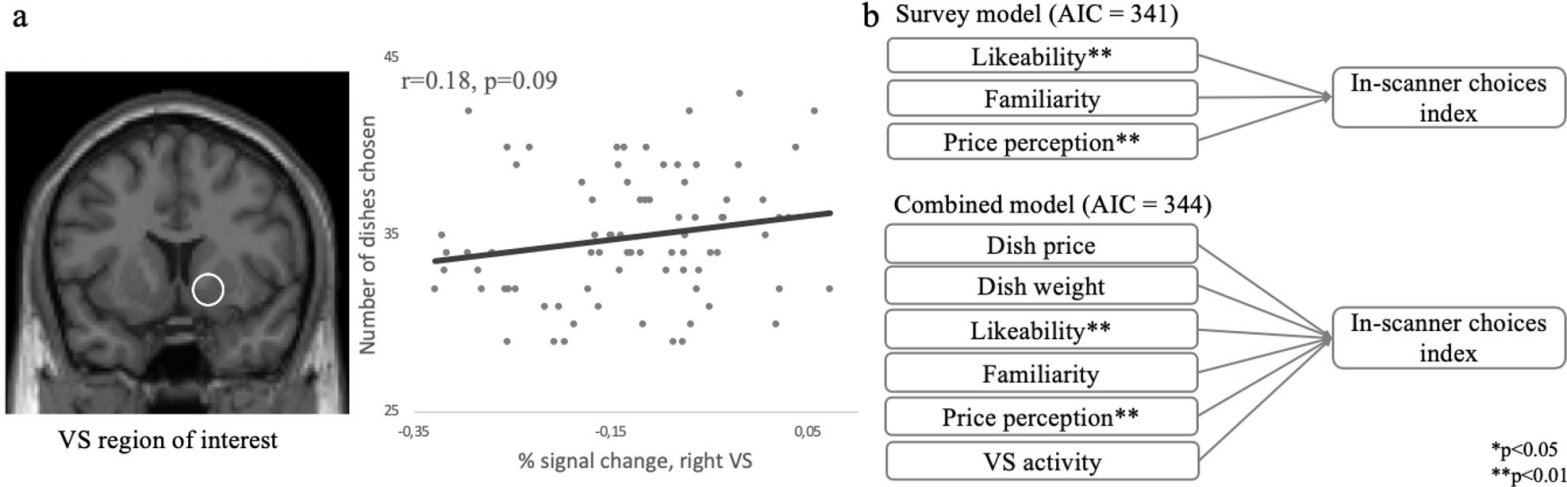
Relationship among VS activity, survey data, dishes’ objective characteristics (dish’s price and weight) and *in-scanner choices index*. (a) The *functional ROI* and the scatterplot indicating *in-scanner choices index* as a function of averaged VS activity. The line represents a linear trend estimate. (b) The diagrams show key results of linear mixed regression models estimated whether objective characteristics of dishes (price and weight), survey metrics (likeability, familiarity, price perception), and VS activity were predictive of *in-scanner choices index*. Akaike information criterion value is given in parenthesis; asterisks indicate significant coefficients (*p < .05, **p < .01).

Additionally, we built separate linear regression models to estimate whether (a) objective characteristics (dish price and weight), (b) survey metrics (likeability, familiarity, price perception), and (c) VS activity (*functional ROI*) were predictive of the *in-scanner choices index* (see Fig 4 and Table 3). During the fMRI session we collected only in-scanner choices and no other behavioral measures, therefore, regression models for the *in-scanner choices index* contained no (in-scanner) behavioral predictor except survey metrics that were collected after the fMRI session. The AIC indicated a better fit of *in-scanner Model II*, which combined survey variables (AIC = 341), compared with *in-scanner Model I* (AIC = 410), *in-scanner Model III* (AIC = 426), and *in-scanner Model IV* (AIC = 344). Interestingly, VS activity alone (*in-scanner Model III*) was not significantly associated with the *in-scanner choices index*, while a combination of VS activity, participants’ preferences, price perception, and objective product characteristics (*in-scanner Model IV*) explained 69% of the variance in the *in-scanner choices index*.

**Table 3:** Results of the hierarchical linear regression models predicting individual *in-scanner choices index* using objective characteristics of dishes (price, weight), survey or/and neuroimaging data for the *functional ROI*.

Predictor	Model				
	Objective characteristics (*In-scanner Model I*)	Survey (*In-scanner* Model II)	Brain activity (*In-scanner* -Model III)	Combined (*In-scanner* Model IV)	
*Intercept*	16.03(1.35)**	10.55(0.23)**	10.59(0.68**		9.78(1.38)**
*Dish price*	-0.01(0.01)**				0.01(0.03)
*Dish weight*	-0.01(0.01)				-0.01(0.02)
*Likeability*		6.54(0.73)**			6.44(0.73)**
*Familiarity*		0.88(1.12)			0.80(1.14)
*Price perception*		-3.67(0.38)**			-4.09(0.76)**
functional ROI:					
*Right VS* ^ⅰ^			-1.75(1.76)		-1.06(1.03)
R^2^ marginal	0.19	0.69	-0.01		0.69
R^2^ сonditional	0.32	0.69	0.13		0.69
AIC	410	341	426		344

^ⅰ^ *functional ROI*

The table presents standardized coefficients with standard error in brackets. +indicates p-value<0.1; *indicates p-value<0.05

**indicates p-value<0.01

Finally, we built separate linear regression models to estimate, whether and which of the ROIs ((a) *functional-ROI*, (b) *meta-analysis-based ROIs* and (c) *neuroforecasting-based* ROIs) improves better prediction of *sales index*. Results are presented in Table 4.

**Table 4:** Results of the linear regression models predicting *sales index* using different ROIs for neuroimaging data. The table illustrates an advantage of the regression models that include neuroimaging data.

Predictor		Model		
	Combined: objective characteristics & in-scanner choice & survey (combined Model VI)	Combined: objective characteristics & in-scanner choice & survey & functional ROI (combined Model V)	Combined: objective characteristics & in-scanner choice & survey & meta-analysis-based ROIs (combined Model VII)	Combined: objective characteristics & in-scanner choice & survey & neuroforecasting study-based ROIs (combined Model VIII)
*Intercept*	24.77(11.26)*	19.61(10.85)+	27.81(10.75)*	23.69(10.48)*
*Dish price*	-0.01(0.03)	-0.01(0.02)	-0.01(0.02)	-0.01(0.02)
*Dish weight*	0.03(0.02)	0.03(0.02)	0.02(0.02)	0.02(0.02)
*Choice in scanner*	13.42(9.57)	15.21(9.23)^+^	16.16(8.92)^+^	11.33(8.70)
*Likeability*	5.77(8.32)	4.97(7.99)	4.82(7.76)	6.01(7.69)
*Familiarity*	-16.94(8.08)*	-15.55(7.79)*	-19.23(7.78)**	-16.53(7.38)*
*Price perception*	3.24(5.98)	0.85(5.80)	3.72(5.65)	1.11(5.54)
functional ROI:			
*Right VS* ^ⅰ^		19.42(7.06)**	
meta-analyses-based ROIs:			
*Left VS* ^ⅰⅰ^			-11.44(15.27)
*Right VS* ^ⅰⅰ^			41.95(14.37)**
*vmPFC*			-30.26(15.55)+
neuroforecasting study-based ROIs :			
*Left VS* ^ⅰⅰⅰ^				5.33(9.20)
*Right VS* ^ⅰⅰⅰ^				9.73(8.80)
*Left mPFC* ^ⅰⅰⅰ^				-26.47(12.63)*
*Right mPFC* ^ⅰⅰⅰ^				-0.37 (11.34)
*Left AI* ^ⅰⅰⅰ^				-4.14(7.71)
*Right AI* ^ⅰⅰⅰ^				9.66(7.68)
*Left Am* ^ⅰⅰⅰ^				12.56(6.77)^+^
*Right Am* ^ⅰⅰⅰ^				11.12(9.04)
R^2^ marginal	0.09	0.17	0.16	0.17
R^2^ сonditional	0.28	0.33	0.36	0.39
AIC	657	652	651	653
*LRStat*		7.1	11.7	19.8
*p-value*		0.007	0.009	0.01

The table presents standardized coefficients with standard error in brackets. +indicates p-value<0.1; *indicates p-value<0.05

**indicates p-value<0.01.

^ⅰ^ functional ROIs

^ⅰⅰ^ meta-analyses-based ROIs

^ⅰⅰⅰ^ neuroforecasting study-based ROIs

## Discussion

Our findings further support the view that neural activity underlying reward processing could predict decisions at the aggregate level. In an fMRI study, we analyzed the VS activity of 22 participants while they observed images of dishes from the new menu of a popular restaurant chain. This activity significantly correlated with the global (one-year) sales of the restaurant chain. Linear regression modeling indicated that the combination of neural data, choice, and survey data can account for global sales better than in-scanner choices or survey data alone. Taken together, our results offer modest support for a number of neuroforecasting findings, demonstrating that the VS activity of a group of participants may forecast the behavior of an independent group [1–10].

Despite a growing number of neuroforecasting studies, the overall number of such studies—and the scope thereof—remains limited (for a review, see [12]). To the best of our knowledge, the present research is the first study to demonstrate that VS responses to primary rewards and images from the new menu of a popular restaurant chain could predict aggregate global sales. In the current study, the VS activity predicting global sales was located in the ROI which was defined based on participants’ preference-related activity. Importantly, in numerous respects, the VS is a rather heterogeneous brain region. First, VS neurons contain different types of dopamine receptors (for a review, see [38, 39]). Second, the VS receives a number of inputs, which often overlap at the population and single-neuron level (for a review, see [40, 41]); however, the subregions of the VS highly differ in terms of the topographical organization of inputs from the temporal and frontal lobes (for a review, see [42, 43]). Third, various studies have reported a functional heterogeneity within the VS; for example, the regional distribution of activity during the anticipation and outcome phases of decisions differs in the VS [44]. Finally, the activations to rewards versus neutral stimuli, to high versus low amount of reward, and to reward versus loss reveal a regional heterogeneity in the VS as well [44], and different subregions of the VS showed code reward magnitude and probability [45]. Overall, the VS is heterogeneous at the neurochemical, histological, anatomical, and functional levels, leading to the speculation that perhaps the ROIs in the VS in neuroforecasting studies should be relatively reward/domain-specific.

The VS is implicated not only in the anticipation of reward delivery [46], but also in the processing of rewarding stimuli [47, 48], including reward value learning [49] and hedonic responses [50, 51]. A recent fMRI study showed that the different subregions of the VS are involved in the motivational and hedonic processing of rewards [52]. However, reward-related hedonic experience has been most consistently associated with the activity of the vmPFC rather than the VS [53]. A meta-analysis showed that, indeed, vmPFC was recruited only during the reward outcome, likely representing the value of the reward received [28]. Thus, our findings that VS activity forecasts the aggregate choices for primary rewards further support previous findings emphasizing the role of the VS in anticipation of rewards, including the processing (evaluation) of products during purchasing decisions [54].

Further, we found a significant correlation of aggregate sales of food items with VS activity in the ROI which was defined based on the preference-related activity of our participants. The hierarchical linear regression model—which combined VS activity, behavior, and survey data and objective product characteristics—explained approximately 69% of the variance in individual in-scanner choices and 33% of the variance in aggregate population behavior, here as indicated by the *sales index*. This is comparable with previous neuroforecasting studies. For example, a similar analysis conducted by Tong et al. explained 51% of the population’s behavior (see Table 1 in [5]). A difference in explanatory power of neuroforecasting studies can be explain by various reasons, which are hard to standardize in each neuroimaging study: from the ROI selection criteria till the sensitivity of the indexes that are used to estimate aggregate choices.

The lower accuracy in predicting behavior at the population level when compared with predicting participants’ behavior in the current study can be related to the fact that global sales are affected by numerous factors. These factors are notoriously difficult to control for. Importantly, the model that included VS activity indicated a better fit with *sales index* than the model that included only behavior, objective, and survey data, thereby supporting previous findings (i.e., [3, 4]). Thus, our results further demonstrate that aggregate behavior can be better predicted by a combination of “hidden” neurophysiological variables and behavioral or self-reported measures as compared to the latter on its own.

As predicted by the affect-integration-motivation (or AIM) framework, the “affective” VS activity may broadly generalize broadly across individuals, thereby revealing “hidden information” regarding mass preferences [55]. Furthermore, the AIM framework suggests that affective responses—that underlie our choices—are most likely to generalize across individuals and predict aggregate choices [15]. In this framework, reward-related activity in the VS is integrated with other contexts in the vmPFC, dorsomedial prefrontal cortex, and dorsal striatum. The AIM framework suggests that affective neural circuitry (VS and insula) may indicate greater choice consistency across individuals, while integrative components may confer greater choice consistency within individuals [56]. Importantly, functional heterogeneity of the VS may suggest that neuroforecasting studies must carefully select ROIs, adjusting them to the type of reward that is used in the study. For example, an extensive meta-analysis showed overlapping activation between reward and loss anticipation, but the portion of the right VS corresponding to the nucleus accumbens shell was reliably active during anticipation of reward but not with that of loss [28]. Therefore, subregions of the VS forecasting the aggregate popularity of comedy might differ from subregions of the VS underlying the forecasting of the popularity of horror films. Our results only partially support the domain-general role of the VS in reward processing, showing that a functionally defined VS ROI can be most effective in neuroforecasting aggregate choice for primary rewards. Interestingly, only left insular cortex activity significantly predicted the *in-scanner choices index* in the combined supplementary models (S5 Table in S1 File), while activity in the left vmPFC was negatively associated with the *sales index* (Table 4). Thus, in our study, affective components of choice-related neural activity reflected both choice consistency across individuals (VS) and choice consistency within individuals (insular cortex). Future studies should test other ROI selection algorithms (e.g., using various functional localizers) to check the advantage of neural activity that is truly specific for particular types of aggregate choices.

It is also worth noting that activity in the right (but not left) VS was significantly associated with *sales index*. An activation likelihood estimation meta-analysis of 50 fMRI studies, which used the Monetary Incentive Delay Task, identified reliable activity, particularly in the right VS, to reward anticipation relative to loss anticipation [28]. A seminal study [23] demonstrated the peak activity for the expected subjective value of food in the right VS, while the activity for the expected subjective value of money peaked in the left VS. In addition, some studies implicated the right VS into eating disorders [29] or showed stronger right VS activity related to the behavioral preferences for food flavors [30].

We also found that activity in the left vmPFC was negatively associated with the *sales index* (Table 4). Because the participants in our study preferred unfamiliar dishes, context-dependent vmPFC activity could be modulated by familiarity with dishes. Previously, stronger vmPFC activity has been reported when the participants imagined personal future events within a familiar contextual setting compared with an unfamiliar contextual setting [57, 58]. Further studies should test the role of the left vmPFC in food processing.

The current study has several limitations that must be acknowledged and considered for future research. For fMRI scanning, we invited only those participants who met a number of criteria and matched an average customer profile which we received from the marketing department of the restaurant chain. Thus, the generalizability of the results of this study must be made with caution in the context of the general population. In the current study, one of the participant’s choices was randomly selected, and a voucher for this dish was provided for free. Thus, price information could not affect the participants’ payoff, which may limit the effect of price on the participants’ choices. The ecological validity of the future neuroforecasting findings might benefit from further testing of other study designs by using the participants’ personal records of dishes purchased in the restaurant chain, enabling the participants to spend their own budget to purchase dishes during the scanning session, delivering the selected dish immediately after the scanning session, and so forth. Similarly, fMRI settings affect participants’ body position, such as the restaurant interior and social contexts (friends, other customers, waiters, etc.). In real life, making food choices may take a different amount of time than that taken in our study and may differ among different dishes. Here, the authors extract brain activity from the ROI was defined within the same task. This is especially problematic when the data are used to model in-sample behavior. Overall, further studies are clearly needed in order to extend our findings to real-life decisions. Moreover, additional information regarding seasonality, ad campaigns, current dish promotions, and in-restaurant activities may improve the predictive power of neuroforecasting.

Overall, our results demonstrated an ability to neuroforecast aggregate choices of primary rewards. The hierarchical linear regression model demonstrated a significant association of VS activity with aggregate sales. Notably, a combination of neural, behavioral, survey data and objective product characteristics explained aggregate customers’ choices better than each predictor individually. Thus, our results extend the neuroforecasting approach to the domain of dining, thus making an additional step in the verification and development of the new field of neuroforecasting.

## Supporting information

S1 File (DOCX)

S1 Data Contains following data: (1) dishes.data (weight, price, category); (2) indexes.mat (*sales index and in-scanner choices index); (3)* Individual_data.mat (survey, behavioral and fMRI data, including all ROIs, for each subject); (4) control regions data.
(ZIP)
