# Correction: The Prediction of Market-level Food Choices by the Neural Valuation Signal

**DOI:** 10.1371/journal.pone.0295161

**Published:** 2023-12-28

**Authors:** Andrew Kislov, Anna Shestakova, Vadim Ushakov, Mario Martinez-Saito, Valeria Beliaeva, Olga Savelo, Aleksey Vasilchuk, Vasily Klucharev

An additional affiliation is missing for the eighth author. Vasily Klucharev is also affiliated with HSE Graduate School of Business, Moscow, Russia.

In the Funding statement, the grant number from the funder International Laboratory for Social Neuroscience of the Institute for Cognitive Neuroscience HSE is listed incorrectly. The correct RF Government Grant Number is 075-15-2022-1037.

There is error in the formatting of Table 4, and it is not possible to make the correct conclusions from the table. Please see the correct Table 4 here.

**Table 4:** Results of the linear regression models predicting sales index using different ROIs for neuroimaging data. The table illustrates an advantage of the regression models that include neuroimaging data.

Predictor		Model		
	Combined: objective characteristics & in-scanner choice & survey (combined Model VI)	Combined: objective characteristics & in-scanner choice & survey & functional ROI (combined Model V)	Combined: objective characteristics & in-scanner choice & survey & meta-analysis-based ROIs (combined Model VII)	Combined: objective characteristics & in-scanner choice & survey & neuroforecasting study-based ROIs (combined Model VIII)
*Intercept*	24.77(11.26)*	19.61(10.85)+	27.81(10.75)*	23.69(10.48)*
*Dish price*	-0.01(0.03)	-0.01(0.02)	-0.01(0.02)	-0.01(0.02)
*Dish weight*	0.03(0.02)	0.03(0.02)	0.02(0.02)	0.02(0.02)
*Choice in scanner*	13.42(9.57)	15.21(9.23)^+^	16.16(8.92)^+^	11.33(8.70)
*Likeability*	5.77(8.32)	4.97(7.99)	4.82(7.76)	6.01(7.69)
*Familiarity*	-16.94(8.08)*	-15.55(7.79)*	-19.23(7.78)**	-16.53(7.38)*
*Price perception*	3.24(5.98)	0.85(5.80)	3.72(5.65)	1.11(5.54)
**functional ROI:**				
*Right VS* ^**ⅰ**^		19.42(7.06)**		
** meta-analyses-based ROIs:**				
*Left VS* ^**ⅰⅰ**^			-11.44(15.27)	
*Right VS*^**ⅰⅰ**^)			41.95(14.37)**	
*vmPFC*			-30.26(15.55)+	
**neuroforecasting study-based ROIs :**				
*Left VS* ^**ⅰⅰⅰ**^				5.33(9.20)
*Right VS* ^**ⅰⅰⅰ**^				9.73(8.80)
*Left mPFC* ^**ⅰⅰⅰ**^				-26.47(12.63)*
*Right mPFC* ^**ⅰⅰⅰ**^				-0.37 (11.34)
*Left AI* ^**ⅰⅰⅰ**^				-4.14(7.71)
*Right AI* ^**ⅰⅰⅰ**^				9.66(7.68)
*Left Am* ^**ⅰⅰⅰ**^				12.56(6.77)^+^
*Right Am* ^**ⅰⅰⅰ**^				11.12(9.04)
R^2^ marginal	0.09	0.17	0.16	0.17
R^2^ сonditional	0.28	0.33	0.36	0.39
AIC	657	652	651	653
*LRStat*		7.1	11.7	19.8
*p-value*		0.007	0.009	0.01

The table presents standardized coefficients with standard error in brackets. +indicates p-value<0.1; *indicates p-value<0.05; **indicates p-value<0.01.

i functional ROIs

ii meta-analyses-based ROIs

iii neuroforecasting study-based ROIs
