# Supplementary material for: The prediction of market-level food choices by the neural valuation signal
Source: PLoS One. 2023 Jun 2;18(6):e0286648. doi: 10.1371/journal.pone.0286648 (PMC10237376; doi:10.1371/journal.pone.0286648)
Supplement: S1 File — (DOCX) [file pone.0286648.s001.docx]

**Supplementary materials**

**The Prediction of Market-level Food Choices by the Neural Valuation Signal**

Andrew Kislov, Anna Shestakova, Vadim Ushakov, Mario Martinez-Saito, Valeria Beliaeva, Olga Savelo, Aleksey Vasilchuk, Vasily Klucharev

**Contents**

[Whole-brain analysis 2](#_Toc132053418)

[Descriptive statistics for in-scanner behavior and survey metrics 4](#_Toc132053419)

[Correlation between VS activity during the initial presentation of the dishes and individual in-scanner decisions 4](#_Toc132053420)

[The results of supplementary hierarchical linear mixed regression models that predicted the *sales index* and *in-scanner choices index* using behavioral, survey, and/or neuroimaging data 5](#_Toc132053421)

[Differential neural activity (high vs. low demand) for different ROIs 10](#_Toc132053422)

[The results of control supplementary hierarchical linear mixed regression models that predicted the *sales index* *and in-scanner choices index* using behavioral, survey, and/or neuroimaging data, and control ROIs (posterior insula and occipital cortex) 12](#_Toc132053423)

[List of dishes used in the fMRI study 17](#_Toc132053424)

[Supplementary references 20](#_Toc132053425)

## Whole-brain analysis

Maps of *t*-statistics for the regressor of interest were transformed into maps of *z*-scores, co-registered with structural maps, spatially normalized by warping to MNI space, and resampled as 2-mm^3^ voxels. Each group map was initially voxel-wise thresholded (at *p* < 0.001), then cluster thresholded (cluster size > 12 contiguous 2-mm^3^ voxels) to yield a corrected threshold for detecting whole-brain activation (*p* < 0.05 FWE-corrected, derived with 15000 Monte Carlo iterations using SPM12; for the same approach, see (1)). Next, we were applied the metanalysis-based mask that included regions, which showed more positive than negative effects of the subjective value (2).

*Dish preference contrast (i.e., the “Picture” stage of the trial).* The whole-brain analysis included two event-related regressors of interest: (1) onset of the pictures of dishes with relatively high likeability (i.e., rated higher than “3” on the on the 5-point “Do you like it?” Likert scale); and (2) onset of the pictures of dishes with relatively low likeability (i.e., rated lower than “3” on the 5-point “Do you like it?” scale). The analysis also included eight regressors of no interest.(six indexed residual motion, and two indexed activity associated with cerebrospinal fluid and white matter intensity) (3). *Dish preference* *contrast* was calculated by comparing neural activity evoked by the dishes with relatively high likeability versus neural activity evoked by the dishes with relatively low likeability.

*Dish choice contrast (i.e., the “Price” stage of the trial).* The analysis included two event-related regressors of interest: (1) onset of the price for dishes that the participants chose to eat at the end of the study; and (2) onset of the price for dishes that participants did not choose to eat at the end of the study. The analysis also included eight regressors of no interest, that indexed residual motion and activity associated with cerebrospinal fluid and white matter intensity. *Dish choice contrast* was calculated by comparing neural activity evoked by the price of the chosen dishes versus neural activity evoked by the price of the dishes that were not chosen.

Table S1 summarizes the results of the whole-brain analysis. The whole brain analysis of the *dish preference* contrast yielded no significant result. Importantly, we observed that the uncorrected data (thresholded at p < 0.001) demonstrated right VS activity (Figure S1). For the *dish choice* contrast, we observed significant vmPFC activation for the dishes that were chosen by subjects compared to the dishes that were not chosen by subjects: Z = 3.93; p FWE-corrected cluster-level = 0.001 (Figure S2).


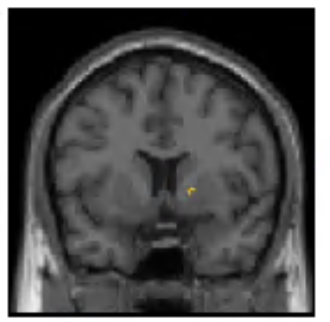


**Figure S1**. Brain activity to the onset of pictures (dishes) that was associated with participants’ preferences (*dish preference* contrast).

For illustrative purposes, the uncorrected data thresholded at p < 0.001 is shown in Figures S1 and S2.


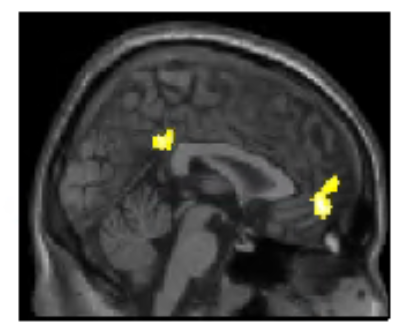


**Figure S2.** Brain activity to the price of the dish that was associated with in-scanner choices (*dish choice* contrast)

**Table S1.** Results of the whole-brain analysis

| Contrast | Region | X | Y | Z | Peak Z | P (FWE-corrected cluster-level) |
| --- | --- | --- | --- | --- | --- | --- |
| *Dish preference* |  |  | none |  |  |  |
| *Dish choice* | Ventromedial prefrontal cortex | 2 | 52 | -6 | 4.3 | 0.001 |

## Descriptive statistics for in-scanner behavior and survey metrics

*Descriptive statistics.* We calculated several behavioral and survey indexes for each dish: *in-scanner choices* (percentage of people who selected the dish during the fMRI session), *likeability* (percentage of people who selected a rating of more than “3” on the on the 5-point Likert scale “Do you like it?”), *familiarity* (percentage of people who answered “Yes” to the question “Have you tried this dish before?”), and *price perception* (percentage of people who selected a rating of more than “3” on the on the 5-point Likert “Are you satisfied with the price of this dish?” scale). The descriptive statistics are presented in Table S2.

**Table S2.** Descriptive statistics for in-scanner behavior and survey metrics

|  | *In-scanner choices*  (%) | *Likeability* (%) | *Familiarity* (%) | *Price perception* (%) |
| --- | --- | --- | --- | --- |
| Min | 29 | 62 | 28 | 55 |
| Max | 43 | 94 | 39 | 99 |
| Average | 34.9 | 79.5 | 34.3 | 76.9 |
| Median | 34 | 79 | 34 | 77 |
| StD | 3.4 | 7 | 2.2 | 8.9 |

## Correlation between VS activity during the initial presentation of the dishes and individual in-scanner decisions

*Correlation analysis.* We calculated correlations between the *in-scanner choices* *index* and brain activity in the pre-defined the *functional ROI*, *meta-analyses-based ROI*, and *neuroforecasting study-based ROI* during the initial presentation of the dishes (i.e., the “Picture” stage of the trial). The correlations between the *in-scanner choices* *index* and average VS activity in the *functional ROI*, *meta-analyses-based ROI*, and *neuroforecasting study-based ROI* were not significant. These results are summarized in Table S3.

**Table S3.** Correlation between VS activity during the initial presentation of the dishes (i.e., the “Picture” stage of the trial) and *in-scanner choices index* (aggregated in-scanner choices)

|  | ROI | MNI coordinates | Correlation with *in-scanner choices index* (r) | p-value |
| --- | --- | --- | --- | --- |
| Right VS | *functional ROI* | 18 8 2 | 0.18 | 0.09 |
| Right VS | *meta-analysis-based ROI* (based on (2) | 12 10 -6 | 0.02 | 0.87 |
| Right VS | *neuroforcasting study based* (based on (1)) | 11 15 -6 | 0.08 | 0.46 |

## The results of supplementary hierarchical linear mixed regression models that predicted the *sales index* and *in-scanner choices index* using behavioral, survey, and/or neuroimaging data

*Supplementary analysis of the sales index.* We adopted MNI coordinates from a previous neuroforecasting paper (1) and meta-analysis (2) and constructed two additional models, which included VS, vmPFC, anterior insula, and amygdala activity (Table S6), to further explore the neural predictors of the *sales index*:

1. *Model S1* included brain activity from ROIs in the right/left VS and vmPFC as defined by coordinates from the meta-analysis (2) as predictors.
2. *Model S2* included brain activity from the ROIs in the right/left VS, right/left vmPFC, right/left anterior insula, and right/left amygdala as defined based on coordinates from a previous neuroforecasting study (1).

Table S4 summarizes the results of Model S1 and S2.

**Table S4.** Results of supplementary linear mixed regression models predicting the *sales index* using brain activity from ROIs as defined based on the meta-analysis or a previous neuroforecasting study.

| Predictor |  | **Model** | | | | | | |
| --- | --- | --- | --- | --- | --- | --- | --- | --- |
|  |  |  | | Brain activity: meta-analysis-based ROIs  (Model S1) | | Brain activity: neuroforecasting study-based ROIs  (Model S2) | | |
|  |  |  | |  | |  |  |  |
| *Constant term* |  |  | | 30.90(3.73)** | |  | 26.69(3.16)** |  |
| **meta-analyses-based ROIs:** | |  |  | |  | | | |
| *Left VS***^ⅰ^** |  |  | | -1.96(17.15) | |  |  |  |
| *Right VS***^ⅰ^** |  |  | | 36.63(16.17)* | |  |  |  |
| *vmPFC***^ⅰ^** |  |  | | -29.16(16.80)^+^ | |  |  |  |
|  |  |  | |  | |  |  |  |
| **neuroforecasting study-based ROIs:** |  |  | |  | |  | | |
| *Left VS***^ⅰⅰ^** |  |  | |  | | 7.09(9.95) | | |
| *Right VS***^ⅰⅰ^** |  |  | |  | | 4.77(9.10) | | |
| *Left mPFC***^ⅰⅰ^** |  |  | |  | | -37.69(13.46)** | | |
| *Right mPFC***^ⅰⅰ^** |  |  | |  | | 10.64(12.05) | | |
| *Left AI***^ⅰⅰ^** |  |  | |  | | 0.40(8.28) | | |
| *Right AI***^ⅰⅰ^** |  |  | |  | | 14.15(8.43)^+^ | | |
| *Left Am***^ⅰⅰ^** |  |  | |  | | 11.44(7.44) | | |
| *Right Am***^ⅰⅰ^** |  |  | |  | | 7.57(9.78) | | |
|  |  |  | |  | |  | | |
| R^2^ marginal |  |  | | 0.06 | | 0.1 | | |
| R^2^ сonditional |  |  | | 0.19 | | 0.27 | | |
| AIC |  |  | | 659.3 | | 657.92 | | |

The table presents standardized coefficients with standard error in brackets. +indicates p-value<0.1; *indicates p-value<0.05; **indicates p-value<0.01

^ⅰ^ meta-analyses-based ROIs

^ⅰⅰ^ neuroforecasting study-based ROIs

*Supplementary analysis of in-scanner choices* *index*. Additionally, we used the same approach to examine which variables determine the *in-scanner choices* *index*:

1. *Model S3* included brain activity from ROIs in the right/left VS and vmPFC as defined by coordinates from the meta-analysis (2).
2. *Model S4* included brain activity from the ROIs in the right/left VS and vmPFC as defined by coordinates from the meta-analysis (2), objective characteristics of the dishes (price and weight of each dish) and survey data (participants’ preferences: such as whether participants liked the dish, were familiar with the dish, and considered the dish’ price high or low) .
3. *Model S5 i*ncluded brain activity from the ROIs in the right/left VS, right/left vmPFC, right/left anterior insula, and right/left amygdala as defined by coordinates from the previous neuroforecasting study (1).
4. *Model S6* included brain activity from the ROIs in the right/left VS, right/left vmPFC, right/left anterior insula, and right/left amygdala as defined by coordinates from the previous neuroforecasting study (1), objective characteristics of the dishes and survey data.
5. *Model S7* included only objective characteristics of the dishes and survey data as predictors.

**Table S5.** Results of the supplementary linear regression models predicting individual *in-scanner choices index* using objective characteristics of dishes (price, weight), survey data or/and neuroimaging data.

| Predictor |  |  | **Model** | |
| --- | --- | --- | --- | --- |
|  | Brain activity: meta-analysis-based ROIs  (*In-scanner* Model S3) | Combined: objective characteristics & survey & meta-analysis-based ROIs  (*In-scanner* Model S4) | Brain activity: neuroforecasting study-based ROIs  (*In-scanner* Model S5) | Combined: objective characteristics & survey & neuroforecasting study-based ROIs  (*In-scanner* Model S6) |
| *Constant term* | 10.09(0.87)** | 9.40(1.37)** | 10.60(0.66)** | 9.61(1,41)** |
| *Dish price* |  | 0.01(0.01) |  | 0.01(0.01) |
| *Dish weight* |  | -0.01(0.01) |  | -0.01(0.01) |
|  |  |  |  |  |
| *Likeability* |  | 6.40(0.74)** |  | 6.26(0.78)** |
| *Familiarity* |  | 0.65(1.17) |  | 0.61(1.17) |
| *Price perception* |  | -4.11(0.76)** |  | -4.17(0.78)** |
| **meta-analyses-based ROIs:** |  |  |  |  |
| *Left VS***^ⅰ^** | 0.34(3.94) | -0.90(2.33) |  |  |
| *Right VS***^ⅰ^** | -3.82(3.72) | -1.79(2.21) |  |  |
| *vmPFC* | -3.29(3.86) | -0.33(2.38) |  |  |
| **neuroforecasting study-based ROIs:** |  |  |  |  |
| *Left VS***^ⅰⅰ^** |  |  | -1.06(2.36) | -0.49(1.44) |
| *Right VS***^ⅰⅰ^** |  |  | -1.65(2.16) | -0.12(1.38) |
| *Left mPFC***^ⅰⅰ^** |  |  | -1.21(3.19) | -0.70(2.02) |
| *Right mPFC***^ⅰⅰ^** |  |  | 0.24(2.86) | -0.61(1.81) |
| *Left AI***^ⅰⅰ^** |  |  | 5.15(1.97)* | 0.77(1.25) |
| *Right AI***^ⅰⅰ^** |  |  | 0.96(2.00) | 0.08(1.20) |
| *Left Am***^ⅰⅰ^** |  |  | -1.39(1.77) | 0.03(1.08) |
| *Right Am***^ⅰⅰ^** |  |  | -1.49(2.32) | -0.56(1.44) |
|  |  |  |  |  |
| R^2^ marginal | 0.12 | 0.68 | 0.15 | 0.67 |
| R^2^ conditional | 0.002 | 0.68 | -0.005 | 0.66 |
| AIC | 429 | 348 | 432 | 370 |

^ⅰ^ meta-analyses-based ROIs

^ⅰⅰ^ neuroforecasting study-based ROIs

Finally, we tested separate linear regression models to evaluate if (a) functional-ROI, (b) meta-analysis-based ROIs or (c) neuroforecasting study-based ROIs better predict *in-scanner choices index.* The results are presented in Table S6.

**Table S6.** The comparison of linear regression models predicting individual *in-scanner choices index* using neural activity at the different ROIs: functional-ROI, meta-analysis-based ROIs or neuroforecasting study-based ROIs

| Predictor |  | **Model** | | |
| --- | --- | --- | --- | --- |
|  | Combined: objective characteristics & survey  (*In-scanner* Model S7) | Combined: objective characteristics & survey & functional ROIs  (*In-scanner* Model IV) | Combined: objective characteristics & survey & meta-analysis-based ROIs  (*In-scanner* Model S4) | Combined: objective characteristics & survey & neuroforecast-ing study-based ROIs  (*In-scanner* Model S6) |
| R^2^ marginal | 0.69 | 0.69 | 0.68 | 0.66 |
| R^2^ сonditional | 0.69 | 0.69 | 0.68 | 0.67 |
| AIC | 344 | 345 | 348 | 357 |
| *LRStat* |  | 1.05 | 1.63 | 2.83 |
| *p-value* |  | 0.3 | 0.6 | 0.9 |

The AIM framework suggests that affective neural circuitry (VS and insula) may indicate greater choice consistency across individuals, while integrative components (vmPFC) may confer greater choice consistency within individuals. However, we can neither confirm nor deny this hypothesis based on our data. Our results only partially support the domain-general role of the VS in reward processing, showing that a functionally defined VS ROI can be most effective in neuroforecasting aggregate choice for primary rewards. Interestingly, only left insular cortex activity significantly predicted the *in-scanner choices index* in the combined supplementary models (Table S5), while activity in the left vmPFC was negatively associated with the *sales index* (Table 4). Thus, in our study, affective components of choice-related neural activity reflected both choice consistency across individuals (VS) and choice consistency within individuals (insular cortex).

**Table S7.** MNI coordinates of ROIs for supplementary models (adopted from(1))

| ROIs | MNI coordinates |
| --- | --- |
| Left VS | -10 14 -6 |
| Right VS | 11 15 -6 |
| Left mPFC | -4 50 1 |
| Right mPFC | 5 50 1 |
| Left anterior insula | -35 28 -8 |
| Right anterior insula | 36 28 -9 |
| Left amygdala | -25 -3 -22 |
| Right amygdala | 25 -2 -22 |

## Differential neural activity (high vs. low demand) for different ROIs

In order to additionally evaluate which period of the trial yielded the most useful signal for forecasting *sales index*, we analyzed and compared time-courses for *high demand* dishes (50% of dishes with the highest *sales index*) versus *low* *demand* dishes (50% of dishes with the lowest *sales index*). We calculated the difference of the percent signal change (*high* vs. *low* demand dishes) for the onsets of Picture, Description and Price stages of the trial (see Table S7). The differential neural response (*high* *demand* vs. *low* *demand* dishes) was calculated for (i) the functional ROI (VS), (ii) neuroforecasting study-based ROIs and (iii) meta-analyses-based ROIs.

**Table S8.** Differential activity (highly demanded dishes vs. dishes with low demand; %signal change) at the VS and mPFC ROIs.

| ROI | Source | MNI coordinates | Picture onset | Description onset | Price onset |
| --- | --- | --- | --- | --- | --- |
| Right VS | functional ROI | 18 8 2 | 0.04* | 0.06* | 0.01 |
| Left mPFC | **neuroforecasting study-based ROIs** | -4 50 1 | -0.04 | 0.06 | 0.03 |
| Left VS |  | -10 14 -6 | -0.02 | 0.03 | 0.05* |
| Right VS |  | 11 15 -6 | 0.01 | 0.01* | 0.04* |
| Right mPFC |  | 5 50 1 | 0.01 | 0.05* | 0.02 |
| Right VS | meta-analyses-based ROIs | 12 10 -6 | 0.02 | 0.02* | 0.02 |
| Left VS |  | -12 12 -6 | -0.01 | 0.03 | 0.04 |
| vmPFC |  | 2 46 -8 | -0.01 | 0.05* | 0.04 |

*indicates p-value<0.05

Table S8 illustrates that the sales-related differential activity at the *functional ROIs* peaked during the Picture and Description stages of the trials, while other ROIs showed more complex patterns of differential activity. Since the sales-related activity highly differed across stages of the trial, we focused on the initial presentation of dishes during the Picture stage. Importantly, pioneering neuroforcasting studies particularly focused on the initial stimuli presentation: on the initial listening period (4) for songs; project description (5) or photos of consumer products (6). Furthermore, in our study, participants were informed that one of their choices will be selected at random and that they will receive a voucher for this dish. The voucher was provided for free. Thus, due to such design price information did not affect participants’ payoff creating an additional argument to focus on the initial neural response during the presentation food items.

## The results of control supplementary hierarchical linear mixed regression models that predicted the *sales index* *and in-scanner choices index* using behavioral, survey, and/or neuroimaging data, and control ROIs (posterior insula and occipital cortex)

*Supplementary linear mixed regression models for aggregate behavior*

To address the measurement error (7) and further explore the neural predictors of the *sales index* and *in-scanner choices index,* we constructed 14 additional models, that included objective characteristics of dishes (price, weight), behavioral, survey and neuroimaging data, including control regions (PI, left OCC, right OCC). Results are summarized in Tables S9-S13. Tables S9 and S11 report models, exploring predictors of the *sales index,* while Tables S10 and S12 report predictors of the *in-scanner choices index.*

Importantly, inclusion of the insular and visual cortex in the analysis as control regions have not changed our major results: supplementary regression models confirmed that VS activity significantly predicted the aggregate *sales index* (Table S9 and S11). Furthermore, activity of the left mPFC and left amygdala also showed a significant relationship with aggregate sales when the control regions were included to the regression model (Table S12). Interestingly, the only significant neural predator of the *in-scanner choices index* was left insular cortex activity (Table S13).

**Table S9.** Results of the linear regression models predicting the *sales index* using objective characteristics of dishes (price, weight), behavioral, survey, or/and neuroimaging data including the functional ROI and control brain regions.

| \| Predictor \|  \| **Model** \| \| \| \| --- \| --- \| --- \| --- \| --- \| \| Brain activity: control regions (Model C-1) \| Brain activity: control regions & functional ROI  (Model C-2) \| \| Combined: objective characteristics & in-scanner choices & survey & control regions & functional ROI  (Model C-3) \| \| *Intercept* \| 24.42(7.96)** \| 22.88(7.65)** \| 18.25(12.58) \| \| \| \| *Dish price* \|  \|  \| -0.01(0.02) \| \| \| \| *Dish weight* \|  \|  \| 0.03(0.02) \| \| \| \| *Choice in scanner* \|  \|  \| 15.70(9.32)^+^ \| \| \| \| *Likeability* \|  \|  \| 4.71(8.00) \| \| \| \| *Familiarity* \|  \|  \| -15.95(7,85)* \| \| \| \| *Price perception* \|  \|  \| 1.70(6.03) \| \| \| \| **control ROIs:** \|  \|  \|  \| \| \| \| *PI* \| 14.40(15.09) \| 6.60(15.14) \| 7.41(13.81) \| \| \| \| *Left OCC* \| -0.41(23.71) \| -0,45(23.13) \| -7.77(21.23) \| \| \| \| *Right OCC* \| -6.99(18.30) \| -10.47(17,97) \| 1.57(16.36) \| \| \| \| **functional ROI:** \|  \|  \|  \| \| \| \| *VS* \|  \| 18.96(8.13)* \| 18.54(7.48)* \| \| \| \|  \|  \|  \|  \| \| \| \| R^2^ marginal \| -0.26 \| 0.04 \| 0.15 \| \| \| \| R^2^ сonditional \| 0.11 \| 0.14 \| 0.29 \| \| \| \| AIC \| 666 \| 663 \| 657 \| \| \| |
| --- | --- | --- | --- | --- | --- | --- | --- | --- | --- | --- | --- | --- | --- | --- | --- | --- | --- | --- | --- | --- | --- | --- | --- | --- | --- | --- | --- | --- | --- | --- | --- | --- | --- | --- | --- | --- | --- | --- | --- | --- | --- | --- | --- | --- | --- | --- | --- | --- | --- | --- | --- | --- | --- | --- | --- | --- | --- | --- | --- | --- | --- | --- | --- | --- | --- | --- | --- | --- | --- | --- | --- | --- | --- | --- | --- | --- | --- | --- | --- | --- | --- | --- | --- | --- | --- | --- | --- | --- | --- | --- | --- | --- | --- | --- | --- | --- | --- | --- | --- | --- | --- | --- | --- | --- | --- | --- | --- | --- | --- | --- | --- |

The table presents standardized coefficients with standard error in brackets. +indicates p-value<0.1; *indicates p-value<0.05; **indicates p-value<0.01

**Table S10.** Results of the linear regression models predicting the *in-scanner choices index* using objective characteristics of dishes (price, weight), behavioral, survey, or/and neuroimaging data including the functional ROI and control brain regions.

| \| Predictor \|  \| **Model** \| \| \| \| \| --- \| --- \| --- \| --- \| --- \| --- \| \| Brain activity: control regions  (Model C-4) \| Brain activity: control regions & functional ROI  (Model C-5) \| \| Combined: objective characteristics & survey & control regions & functional ROI  (Model C-6) \| \| *Intercept* \| 11.45(1.73)** \| 11.62(1.73)** \| 11.12(1.64)** \| \| \| **control ROIs:** \|  \|  \|  \| \| \| \| *PI* \| 1.65(3.35) \| 2.71(3.38) \| -2.06(2.01) \| \| \| \| *Left OCC* \| 1.65(3.35) \| 1.53(5.18) \| 3.84(2.98) \| \| \| *Right OCC* \| 1.69(5.25) \| 1.01(4.02) \| 0.25(2.37) \| \| \| *Dish price* \|  \|  \| 0.01(0.01) \| \| \| *Dish weight* \|  \|  \| -0.01(0.01) \| \| \| *Likeability* \|  \|  \| 6.37(0.73)** \| \| \| *Familiarity* \|  \|  \| 0.76(1.12) \| \| \| *Price perception* \|  \|  \| -4.23(0.77)** \| \| \| **functional ROI***:* \|  \|  \|  \| \| \| \| *VS* \|  \| -2.38(1.82) \| -0.97(1.07) \| \| \|  \|  \|  \|  \| \| \| R^2^ marginal \| -0.35 \| -0.04 \| 0.69 \| \| \| R^2^ сonditional \| 0.11 \| 0.12 \| 0.69 \| \| \| AIC \| 431 \| 431 \| 347 \| \| |
| --- | --- | --- | --- | --- | --- | --- | --- | --- | --- | --- | --- | --- | --- | --- | --- | --- | --- | --- | --- | --- | --- | --- | --- | --- | --- | --- | --- | --- | --- | --- | --- | --- | --- | --- | --- | --- | --- | --- | --- | --- | --- | --- | --- | --- | --- | --- | --- | --- | --- | --- | --- | --- | --- | --- | --- | --- | --- | --- | --- | --- | --- | --- | --- | --- | --- | --- | --- | --- | --- | --- | --- | --- | --- | --- | --- | --- | --- | --- | --- | --- | --- | --- | --- | --- | --- | --- | --- | --- | --- | --- | --- | --- | --- |

The table presents standardized coefficients with standard error in brackets. +indicates p-value<0.1; *indicates p-value<0.05; **indicates p-value<0.01

**Table S11.** Results of the linear regression models predicting the *sales index* using objective characteristics of dishes (price, weight), behavioral, survey, or/and neuroimaging data including neuroforecasting study-based ROIs, meta-analyses-based ROIs and control brain regions.

| Predictor |  | **Model** | | |
| --- | --- | --- | --- | --- |
|  | Brain activity: control regions & meta-analysis-based ROIs  (Model C-7) | Brain activity: control regions & neuroforecasting study-based ROIs  (Model C-8) | Combined: objective characteristics & in-scanner choices survey & control regions & meta-analysis-based ROIs  (Model C-9) | Combined: objective characteristics & in-scanner choices & survey & control regions & neuroforecasting study-based ROIs  (Model C-10) |
| *Constant term* | 29.59(7.93)** | 22.48(7.59)** | 29.43(12.11)* | 22.26(12.33)^+^ |
| *Dish price* |  |  | -0.01(0.02) | -0.01(0.02) |
| *Dish weight* |  |  | 0.02(0.02) | 0.01(0.02) |
| *Choice in scanner* |  |  | 15.94(8.98)+ | 11.86(8.80) |
| *Likeability* |  |  | 4.69(7.78) | 5.75(7.69) |
| *Familiarity* |  |  | -19.79(7.91)* | -17.03(7.38)* |
| *Price perception* |  |  | 4.02(5.84) | 2.01(5.72) |
| **control ROIs:** |  |  |  |  |
| *PI* | 2.51(15.24) | 8.42(14.01) | 4.22(13.93) | 12.96(12.97) |
| *Left OCC* | 13.47(23.09) | 2.34(22.17) | 4.68(20.87) | -2.94(20.72) |
| *Right OCC* | -16.93(18.25) | -13.47(17.15 | -0.28(16.73) | -2.78(16.02) |
| **meta-analyses-based ROIs:** |  |  |  |  |
| *Left VS***^ⅰ^** | 1.75(18.40) |  | -13.25(16.78) |  |
| *Right VS***^ⅰ^** | 35.96(16.85)* |  | 40.64(15.24)** |  |
| *vmPFC***^ⅰ^** | -32.36(17.01)+ |  | -30.62(15.83)+ |  |
| **neuroforecasting study-based ROIs:** |  |  |  |  |
| *Left VS***^ⅰⅰ^** |  | 7.62(10.52) |  | 3.37(9.77) |
| *Right VS***^ⅰⅰ^** |  | 6.38(9.20) |  | 10,21(8.96) |
| *Left mPFC***^ⅰⅰ^** |  | -36.32(13.44)** |  | -24.88(12.66)+ |
| *Right mPFC***^ⅰⅰ^** |  | 8.90(12.33) |  | -2.07(11.61) |
| *Left AI***^ⅰⅰ^** |  | -0.19(8.34) |  | -4.52(7.82) |
| *Right AI***^ⅰⅰ^** |  | 13.87(8.48) |  | 8.92(7.76) |
| *Left Am***^ⅰⅰ^** |  | 11.61(7.38) |  | 12,62(6.74)+ |
| *Right Am***^ⅰⅰ^** |  | 7.88(9.85) |  | 12.53(9.23) |
|  |  |  |  |  |
| R^2^ marginal | 0.03 | 0.08 | 0.14 | 0.16 |
| R^2^ сonditional | 0.17 | 0.25 | 0.34 | .37 |
| AIC | 664 | 663 | 657 | 658 |

The table presents standardized coefficients with standard error in brackets. +indicates p-value<0.1; *indicates p-value<0.05; **indicates p-value<0.01

^ⅰ^ meta-analyses-based ROIs

^ⅰⅰ^ neuroforecasting study-based ROIs

**Table S12.** Results of the linear regression models predicting the *in-scanner choices index* using objective characteristics of dishes (price, weight), behavioral, declarative, or/and neuroimaging data including the neuroforecasting study-based ROIs, meta-analyses-based ROIs and control brain regions.

| Predictor |  | **Model** | | |
| --- | --- | --- | --- | --- |
|  | Brain activity: control regions & meta-analysis-based ROIs  (Model C-11) | Brain activity: control regions & neuroforecasting study-based ROIs  (Model C-12) | Combined: objective characteristics & control regions & meta-analysis-based ROIs  (Model C-13) | Combined: objective characteristics & survey & control regions & neuroforecasting study-based ROIs  (Model C-14) |
| *Constant term* | 10.98(1.78)** | 12.91(1.76)** | 11.04(1.61)** | 11.63(1.69)** |
| *Dish price* |  |  | 0.01(0.01) | 0.01(0.01) |
| *Dish weight* |  |  | -0.01(0.01) | -0.01(0.01) |
| *Likeability* |  |  | 6.41(0.74)** | 6.14(0.77)** |
| *Familiarity* |  |  | 0.48(1.16) | 0.49(1.13) |
| *Price perception* |  |  | -4.2(0.77)** | -4.23(0.77)** |
| **control ROIs:** |  |  |  |  |
| *PI* | 3.74(3.47) | 1.51(3.29) | -1.77(2.08) | -2.29(2.01) |
| *Left OCC* | 1.74(5.25) | 4.76 (5.20) | 3.99(3.01) | 5.60(3.03)^+^ |
| *Right OCC* | 1.2(4.15) | 1.26(4.02) | 0.90(2.48) | 0.09(2.46) |
| **meta-analyses-based ROIs:** |  |  |  |  |
| *Left VS***^ⅰ^** | -1.38(4.18) |  | -2.38(2.50) |  |
| *Right VS***^ⅰ^** | -4.78(3.84) |  | -0.80(2.29) |  |
| *vmPFC***^ⅰ^** | -3.07(3.87) |  | -0.36(2.35) |  |
| **neuroforecasting study-based ROIs:** |  |  |  |  |
| *Left VS***^ⅰⅰ^** |  | -2.22(2.25) |  | -0.86(1.48) |
| *Right VS***^ⅰⅰ^** |  | -1.96(2.16) |  | -0.01(1.36) |
| *Left mPFC***^ⅰⅰ^** |  | -1.09(3.15) |  | -0.65(1.97) |
| *Right mPFC***^ⅰⅰ^** |  | -0.35(2.89) |  | -0.85(1.81) |
| *Left AI***^ⅰⅰ^** |  | 5.47(1.96)** |  | 1.24(1.23) |
| *Right AI***^ⅰⅰ^** |  | 0.49(1.99) |  | -0.19(1.19) |
| *Left Am***^ⅰⅰ^** |  | -1.15(1.73) |  | 0.03(1.05) |
| *Right Am***^ⅰⅰ^** |  | -1.25 (2.31) |  | -0.90(1.42) |
|  |  |  |  |  |
| R^2^ marginal | -0.03 | -0.03 | 0.69 | 0.67 |
| R^2^ сonditional | 0.12 | 0.15 | 0.69 | 0.67 |
| AIC | 433 | 436 | 351 | 358 |

The table presents standardized coefficients with standard error in brackets. +indicates p-value<0.1; *indicates p-value<0.05; **indicates p-value<0.01

^ⅰ^ meta-analyses-based ROIs

^ⅰⅰ^ neuroforecasting study-based ROIs

## List of dishes used in the fMRI study

*Stimuli.* In the current study, we used photos of dishes from a new menu at a popular restaurant chain, “Chaihona №1,” which has over 25 locations in Moscow. We excluded seasonal dishes from the menu. Six dishes were included for a short training session. The dishes (n =78), which were used in the fMRI data analysis, are listed in Table S1.

**Table S13a**. List of dishes used in the fMRI study

| Dish | Category code | | | Price (RUB) |
| --- | --- | --- | --- | --- |
| Aychichuk | | 6 | 320 | |
| Bahor | | 6 | 210 | |
| Balyk Shurpa | | 7 | 475 | |
| Beef stroganoff with mashed potatoes | | 2 | 640 | |
| Bishtak eggplant | | 1 | 470 | |
| Caesar salad with shrimps | | 6 | 630 | |
| Caesar with chicken | | 6 | 490 | |
| California with crab | | 8 | 510 | |
| Carrot cake | | 3 | 450 | |
| Caucasian chicken shash kebab | | 4 | 465 | |
| Chicken tobacco | | 2 | 650 | |
| Chocolate cake | | 3 | 350 | |
| Chuchvara | | 7 | 365 | |
| Chukka | | 8 | 295 | |
| Crunch | | 8 | 475 | |
| De Luxe eel roll | | 8 | 210 | |
| De Luxe salmon roll | | 8 | 615 | |
| Dessert "Pavlova" | | 3 | 440 | |
| Dragon roll | | 8 | 455 | |
| Dudlyash | | 6 | 315 | |
| Dumplings | | 2 | 350 | |
| Four cheeses | | 5 | 490 | |
| Fresh vegetable salad | | 6 | 345 | |
| Fried suluguni with tomato salad | | 1 | 430 | |
| Geisha | | 8 | 365 | |
| Golden sea bass | | 2 | 280 | |
| Greek salad | | 6 | 390 | |
| Grilled chicken thighs | | 4 | 550 | |
| Grilled vegetables | | 4 | 450 | |
| Haravats | | 1 | 295 | |
| Havas | | 6 | 290 | |
| Hazino dorado | | 2 | 280 | |
| Herring with new potatoes | | 1 | 310 | |
| Homemade baklava | | 3 | 275 | |
| Homemade cheeses | | 1 | 295 | |
| Homemade veal cutlet with mashed potatoes | | 2 | 460 | |
| Honey cake | | 3 | 275 | |
| Humo | | 3 | 440 | |
| Ice cream | | 3 | 125 | |
| Kaurma lagman | | 2 | 440 | |
| Kazy in Tatar | | 1 | 395 | |
| Kazy in Uzbek | | 1 | 395 | |
| Khan's borscht | | 7 | 310 | |
| Kok-samsa with apples | | 1 | 395 | |
| Kok-shurpa | | 7 | 285 | |
| Lagman | | 7 | 425 | |
| Lamb kebab | | 4 | 220 | |
| Lazat | | 6 | 325 | |
| Lemon pie | | 3 | 260 | |
| Lentil soup with sun-dried tomatoes | | 7 | 290 | |
| Li-non with banana and strawberry | | 3 | 670 | |
| Lola | | 6 | 750 | |
| Manti with salmon | | 3 | 450 | |
| Mazza | | 2 | 420 | |
| mozzarella salad | | 6 | 570 | |
| Mushroom cream soup | | 2 | 390 | |
| Napoleon cake | | 3 | 265 | |
| Okinawa | | 8 | 550 | |
| Parma pizza | | 5 | 720 | |
| Philadelphia | | 8 | 550 | |
| Pizza Margherita | | 5 | 450 | |
| Pumpkin puree soup | | 7 | 315 | |
| Rashomon | | 8 | 335 | |
| Russian cabbage soup | | 7 | 260 | |
| Salad mix with avocado and strawberries | | 6 | 280 | |
| Salad with tongue and arugula in walnut sauce | | 6 | 450 | |
| Salmon "chaikhansky" | | 2 | 650 | |
| Shawarma with chicken | | 2 | 520 | |
| Shurpa | | 7 | 435 | |
| Soup kharcho | | 7 | 380 | |
| Suimono | | 8 | 300 | |
| Tashkent | | 6 | 330 | |
| Tom Yum | | 7 | 440 | |
| Tovuk-shurpa | | 7 | 320 | |
| Tuzlama shurpa | | 7 | 395 | |
| Vareniki | | 2 | 300 | |
| Vegetable mix | | 1 | 690 | |
| Zarafshan | | 2 | 610 | |

**Table S13b**. List of eight dish categories used in the fMRI study

| Category name | Category code | | |
| --- | --- | --- | --- |
| Appetizers | | 1 |  |
| Main courses | | 2 |  |
| Desserts | | 3 |  |
| B-B-Q | | 4 |  |
| Pizza | | 5 |  |
| Salads | | 6 |  |
| Soups | | 7 |  |
| Japan food | | 8 |  |

## Supplementary references

1. Genevsky A, Knutson B. Neural affective mechanisms predict market-level microlending. Psychol Sci. 2015;

2. Bartra O, McGuire JT, Kable JW. The valuation system: A coordinate-based meta-analysis of BOLD fMRI experiments examining neural correlates of subjective value. Neuroimage. 2013;

3. Chang C, Glover GH. Effects of model-based physiological noise correction on default mode network anti-correlations and correlations. Neuroimage. 2009;

4. Berns GS, Moore SE. A neural predictor of cultural popularity. J Consum Psychol. 2012;

5. Genevsky A, Yoon C, Knutson B. When brain beats behavior: Neuroforecasting crowdfunding outcomes. J Neurosci. 2017;

6. Tusche A, Bode S, Haynes JD. Neural responses to unattended products predict later consumer choices. J Neurosci. 2010;30(23):8024–31.

7. Webb R, Mehta N, Levy I. Assessing consumer demand with noisy neural measurements. J Econom [Internet]. 2021;222(1):89–106. Available from: https://doi.org/10.1016/j.jeconom.2020.07.028
