# Supplementary material for: The prediction of market-level food choices by the neural valuation signal
Source: PLoS One. 2023 Jun 2;18(6):e0286648. doi: 10.1371/journal.pone.0286648 (PMC10237376; doi:10.1371/journal.pone.0286648)
Supplement: S1 Data — Contains following data: (1) dishes.data (weight, price, category); (2) indexes.mat (sales index and in-scanner choices index); (3) Individual_data.mat (survey, behavioral and fMRI data, including all ROIs, for each subject); (4) control regions data. (ZIP) [file pone.0286648.s002.zip › Data_for_models_legend.docx]

There are several files we provide:

Dishes_data.mat

Indexes.mat

Individual_data.mat

Control.mat

**Dishes_data.mat** includes:

1. ‘Objective1’ consists of dish price (1st column) and dish weight (2nd column) for each of the dishes
2. ‘Categories’ consists of dishes categories

**Indexes.mat** includes:

1. ‘Sales1000’ consists of the *sales index* for each of the dishes
2. ‘Popularity1 consists of the *in-scanner choices index* for each of the dishes

**Individual_data.mat**: includes:

1. Subject number (1^st^ column)
2. ‘Behavior’ consists choice in scanner (2nd column)
3. ‘Survey’ consists of survey data (3rd column – likeability, 4^th^ column – familiarity, 5^th^ column – price perception)
4. ‘Functional_ROI’ consists VS activity (Functional ROI) (6^th^ column)
5. ‘Meta_analysis_ROI’ consists of Left ventral striatum activity (7^th^ column), Right ventral striatum activity (8 ^th^ column), vmPFC activity (9 ^th^ column).
6. ‘Previous_neuroforecasting_ROI’ consists of Left ventral striatum activity (13^th^ column), Right ventral striatum activity (17 ^th^ column), Left mPFC activity (12 ^th^ column), Right mPFC activity (16 ^th^ column), Left AI activity (10 ^th^ column), Right AI activity (14 ^th^ column), Left Am activity (15 ^th^ column), Right Am activity (12 ^th^ column).

**Control.mat**: includes:

Averaged across participants values of the control regions: posterior insula (1^st^ column), left OCC (2^nd^ column), right OCC (3^rd^ column).
